# Improved terminal sliding mode control based on MPC for LIM applied to linear metro

**DOI:** 10.1038/s41598-025-22191-z

**Published:** 2025-10-27

**Authors:** Samir A. Hamad, Fayez F. M. El-Sousy, Moustafa Magdi Ismail, Mahmoud F. Elmorshedy, Dhafer Almakhles, Mohamed. A. Ghalib

**Affiliations:** 1 Process Control Technology Department, Faculty of Technology and Education, Beni-Suef University, Beni Suef, Egypt; 2 Department of Electrical Engineering, College of Engineering, Prince Sattam bin Abdulaziz University, Al Kharj, 16273 Saudi Arabia; 3 Interdisciplinary Research Center (IRC) for Sustainable Energy Systems, King Fahd University of Petroleum and Minerals, Dhahran, 31261 Saudi Arabia; 4 Electrical Engineering Department, Faculty of Engineering, Minia University, EL-Minya, 61517 Egypt; 5 Renewable Energy Lab, College of Engineering, Prince Sultan University, Riyadh, Saudi Arabia; 6 Electrical Power and Machines Engineering Department, Faculty of Engineering, Tanta University, Tanta, 31733 Egypt

**Keywords:** Linear induction machine (LIM), Finite state-model predictive voltage control (FS-MPVC), Terminal sliding mode control (TSMC), Engineering, Electrical and electronic engineering

## Abstract

To improve the dynamic response of the linear induction machine (LIM) over its complete speed range, this paper introduces a finite state-model predictive voltage control (FS-MPVC) based on terminal sliding mode control (TSMC). First, the TSMC for the speed loop is designed to attain high tracking capability and faster transient response. Second, in terms of the intricate balancing coefficient tuning difficulty and several computation steps of the conventional finite state-model predictive thrust control method, the control objectives are transformed to the primary voltage based on the FS-MPVC method. Furthermore, the TSMC-MPVC is fully studied in detail, covering its design and implementation steps. Ultimately, a 3 kW LIM has undergone comprehensive simulation evaluations by the presented TSMC-MPVC through comparing with the conventional methods at various speeds and loads to verify the effectiveness and viability of the proposed method.

## Introduction

**Fig. 1:**
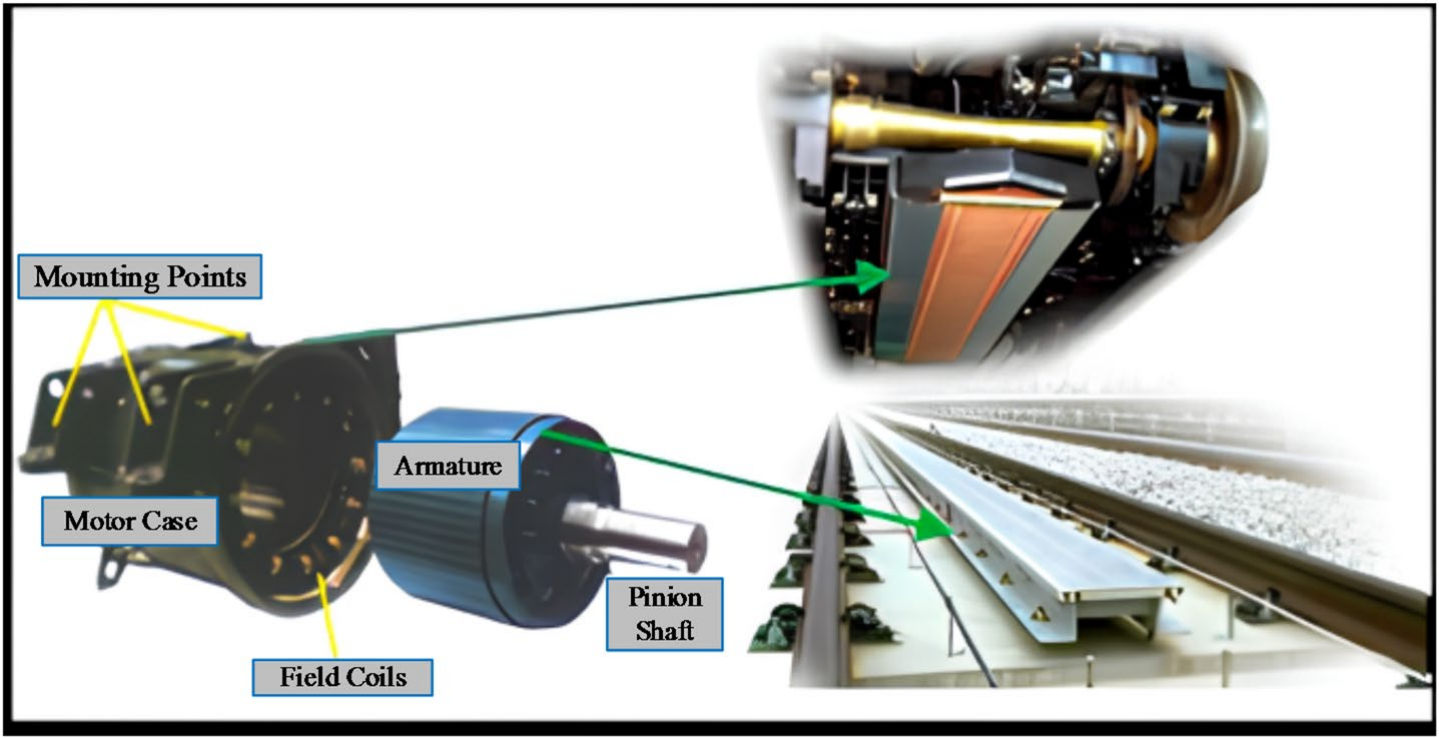
The LIM is represented by RIM.

The linear induction motors (LIMs) are promising option for different industrial applications, particularly light railway^1,2^, due to its ability to get much stronger hill climbing ability, thrust directly capability, smaller turning radius, low maintenance cost, and fast acceleration/deceleration, reduced noise, etc^3^. The LIMs have evolved from the rotary induction motors (RIMs) by adopting the cutting of the main parts, then slicing them open and rolled flat, as shown in Fig. 1. Thus far, in different countries they have been implemented for commercial metro lines systems^4–6^.

Due to the open magnetic circuit, LIM suffers from the problem of longitudinal end effect, in turn, leads to variation in mutual inductance especially with high speed. Such a phenomenon, LIMs have a negative impact on the whole dynamic drive operation, and the LIMs have lower efficiency than the RIMs^7–10^. Generally, because of the primary and secondary of LIMs have high nonlinear characteristics and coupling, the driving performance of conventional control techniques is more challenging on LIMs than on RIMs^11^. There are two main control structures available for the drive systems: field-oriented control, due to the changes in LIM driving system parameters is slower and many parameters are needed and direct thrust control, it has higher ripples for both flux and thrust also has variable switching frequency that caused by the thrust and flux comparator^12–14^. Because of its reliable performance, the field control method is frequently used in RIMs that are applied to traction drive systems. When the rotor flux (d-axis current) stays constant, the traction control drive systems under constant torque will be obtained. The slip frequency (q-axis current), if the aforementioned criterion is met, will proportionately determine the motor torque.

Therefore, a number of research studies have proposed that the traditional direct thrust control (DTC) of the LIM can be used. Three PI regulators had to be properly adjusted for thrust, speed, and flux loop, respectively, in order to use the recommended methods^15–17^.

Thus, in LIM drive systems, increasing focus has been placed on model predictive control (MPC) techniques to address the problems with the conventional DTC and field control methods. Where the advantages of MPC include easier to handle various constraints, online optimization, rapid dynamic response, easy to implement, and so on^18^. The MPC is categorized into two substantial systems, one is finite state-MPC (FS-MPC) and Continuous state-MPC (CS-MPC)^19^. Once more, the FS-MPC is separated into two categories based on the evaluated objective function: (a) FS-Predictive Thrust/Torque Control (FS-PTC), and (b) FS-Predictive Current Control (FS-PCC)^20^.

Generally, the FS-PTC requires to predict the thrust and flux linkage, in which the voltage vector that minimizes the error between the controlled predicted variables with their reference values is selected as the optimal one for applying on the voltage source inverter in the next sampling time. This process needs a balancing coefficient to preference the priorities of the control objectives based on the cost function design^21^.

Undoubtedly, FS-MPC exhibits greater efficiency than the traditional DTC method due to the online evaluation based on a cost function instead of offline switching table and hysteresis comparators. In spite of these abovementioned advantages of the MPC, it has higher computation burden, particularly the tuning of the weighting factors for the FS-PTC approach. Since the FS-PTC needs the balancing coefficient to harmony the control variables of the cost function, the weighting factorless cost functions are proposed in^22–24^ by unifying the cost function dimensions i.e., based on primary flux vector only. However, the proposed method in^25^ the flux item is altered by the thrust item. Because of the items that need to be predicted is reduced, fewer prediction processes are attained, which will improve the computation burden. Additionally, the consuming time in tuning process can be reduced by removing any balancing factor. A brief comparison between different control techniques is summarized in Table 1.

**Table 1:** Summarized comparison between virous control strategies.

Control Strategy	Advantages	Disadvantages
FOC	Lower thrust ripple Lower flux ripple	Slow response Depends on machine parameters Need more coordinate transformations
DTC	Fast response Needs only the primary resistance No need for coordinate transformations	Higher thrust ripple Higher flux ripple Variable switching frequency
FS-PTC	Fast response Lower thrust ripple Lower flux ripple	Depends on the machine parameters

A high-performance LIM drive system can be realized by replacing the PI controller with a robust control approach. Conversely, a sliding mode controller (SMC) is utilized in the drive system due to its robustness, straightforward implementation, and strong adaptability to load variations^26^. Sliding mode control (SMC) is a promising option for transportation applications, offering adaptability to load variations, robustness, insensitivity to unknown parameters and disturbances, and ease of practical implementation. For permanent magnet direct drive systems, a fuzzy sliding mode controller (Fuzzy-SMC) with a smooth switching function is proposed to enhance speed control stability under parameter uncertainties and disturbances. Simulation comparisons with PI and conventional SMC show that Fuzzy-SMC achieves smaller overshoot and superior robustness^27^. A comparative study of first- and second-order sliding mode control for squirrel-cage induction motors using a two-level inverter demonstrates the robustness of SMC in handling uncertainties and disturbances. Results show that FOSMC with a hyperbolic tangent function reduces response time, while SOSMC enhances tracking accuracy and minimizes chattering with fewer control loops^28^.

Therefore, in this paper, the balancing coefficient is removed by adopted the *αβ*-axes primary voltage as a direct control variable in the presented cost function, which is known as FS-PVC. Furthermore, to strengthen the robustness of the PVC method in LIM, a robust terminal sliding mode control (TSMC) is adopted to achieve faster transient response and high tracking ability with lower thrust ripple for the LIM.

The rest of this paper is organized as follows. The dynamic modeling of the LIM drive system is presented in Section II. The FS-PVC method is fully designed and presented in Section III. The speed controller based on TSMC under the FS-PVC strategy is designed for the LIM, which is represented in section IV. Comprehensive simulation results are presented in Sections V, which are fully established that the proposed TSMC based FS-PVC can have stronger anti-disturbance ability, faster convergence speed, smaller thrust and flux ripples, and so on. Finally, conclusions have been drawn.

## Modeling of the LIM

**Fig. 2:**
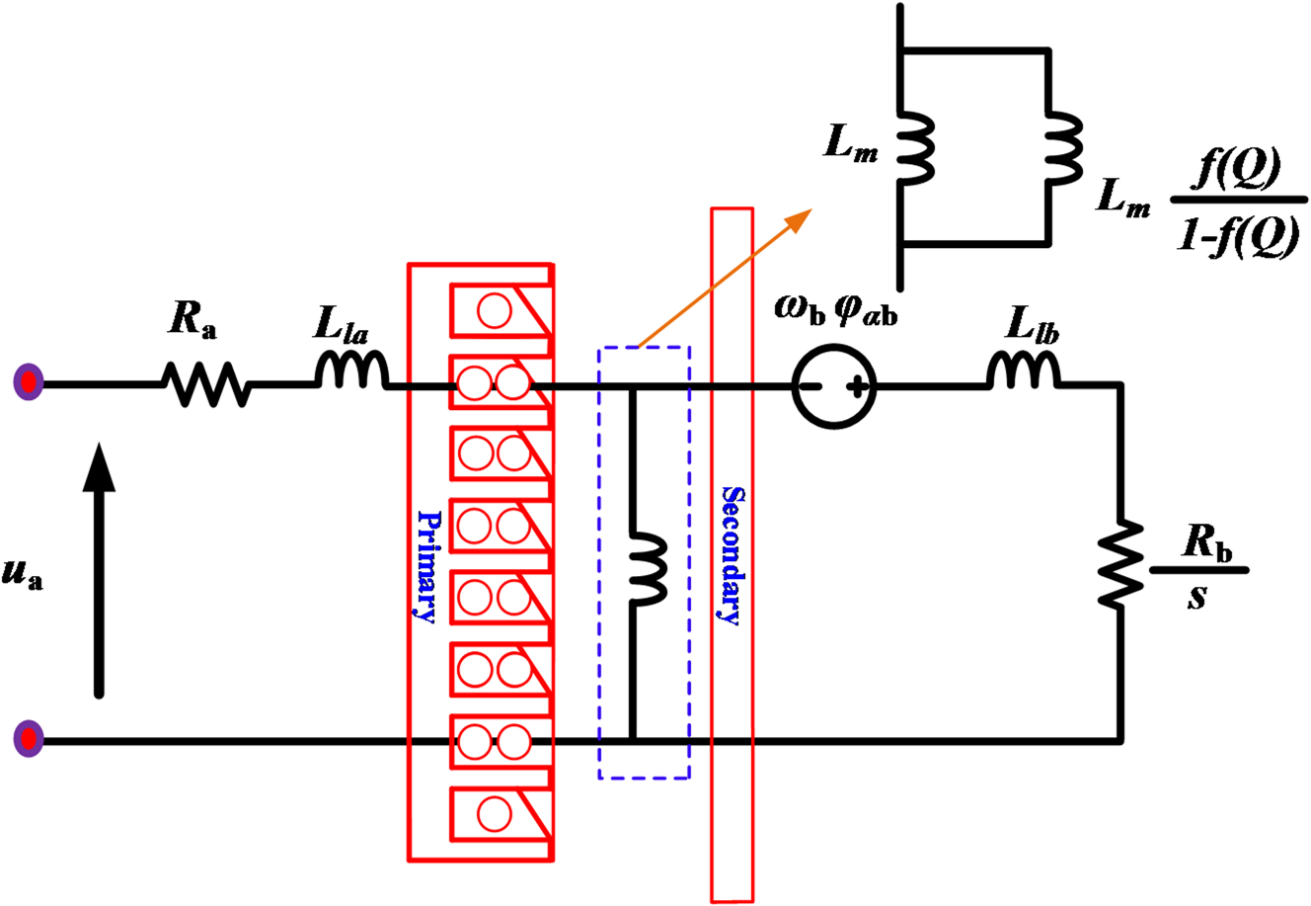
The equivalent circuit of the LIM under *αβ*-axis frame.

Figure 2 illustrates an equivalent circuit of the LIM drive system in the stationary reference frame *αβ*-axis to design its model^29^. According to the aforementioned equivalent circuit, the model of the LIM in stationary frame can be expressed as below:

The voltage equations on the *αβ*-axes can be expressed as

1 $${u_{\alpha \beta {\text{ a}}}}={R_{\text{a}}}{i_{\alpha \beta {\text{ a}}}}+p{\psi _{\alpha \beta {\text{ a}}}} - j{\omega _{\text{a}}}{\psi _{\alpha \beta {\text{ a}}}}$$

2 $${u_{\alpha \beta {\text{ b}}}}={R_b}{i_{\alpha \beta {\text{ b}}}}+p{\psi _{\alpha \beta {\text{ b}}}} - j{\omega _{slip}}{\psi _{\alpha \beta {\text{ b}}}}=0$$

where *a* and *b* refer the primary and secondary parameters of the LIM in the stationary *αβ*-axes for the voltage (*u*), resistance (*R*), current (*i*), and flux (*ψ*), respectively. Additionally, the slip angular velocity *ω*_*slip*_ is defined as the difference between the simultaneous speed *ω*_*a*_ and rotating speed *ω*_*b*_.

The flux equations on the *αβ*-axes are expressed as

3 $${\psi _{\alpha \beta {\text{ a}}}}={L_a}{i_{\alpha \beta {\text{ a}}}}+{L_{meq}}{i_{\alpha \beta {\text{ a}}}}$$

4 $${\psi _{\alpha \beta {\text{ b}}}}={L_b}{i_{\alpha \beta {\text{ b}}}}+{L_{meq}}{i_{\alpha \beta {\text{ b}}}}$$

where *L*_*a*_, and *L*_*b*_, are the primary, secondary leakage inductance parameters; *L*_*meq*_ the equivalent magnetizing inductance modified by the factor *f(Q)*^30^. Then, the electromagnetic thrust is expressed as

5 $${F_e}=\frac{3}{2}\frac{\pi }{\tau }({\psi _{\alpha a}}{i_{\beta a}} - {\psi _{\beta a}}{i_{\alpha a}})$$

where *τ* is denotes as the primary polar length. Then, can be given the mechanical equation by

6 $${F_e}=J(G{\dot {v}_b}+H{v_b})+{F_l}$$

where *J* is the compensate coefficient; *G* is the mass; *H* viscous friction coefficient; *v*_*b*_ is the speed of the LIM; and *F*_*l*_ is the load thrust. The modified magnetizing inductance with correction of the longitudinal edge effects for the LIM, can be updated by the coefficient *f(Q)*, as demonstrated by^31^.

7 $$f(Q)=(1 - exp( - Q))/Q$$

8 $$Q={L_p}{R_b}/{L_{b0}}{v_b}$$

where ${L_a}=~{L_{la}}+{L_{meq}}$ and ${L_b}=~{L_{lb}}+{L_{meq}}$ are the primary and secondary inductances based on the modified magnetizing inductance. The corrected magnetizing inductance is defined as

9 $${L_{meq}}=[1 - f(Q)]{L_m}$$

where *L*_*m*_ is the magnetizing inductance before consideration of the end effect.

## Finite state-model predictive control for LIM drive system

In this section, the finite state-model predictive control is designed for the FS predictive based on voltage control (FS-PVC).

### Finite state model predictive based on voltage control

The thrust and primary flux terms, which have different units, were included in the traditional cost function of the FS-PTC that is being provided. Various dimensions present certain difficulties for thrust and flux error optimization. The predictive voltage control (PVC) approach uses primary voltage exclusively for prediction, minimizing thrust and flux ripples and cutting down on computation time. The cost function in the PVC is applied without the use of a balancing coefficient based on the primary voltage. The thrust and primary flux reference values are utilized to derive the voltage reference using the method described, as shown by

10 $$\vec {V}_{a}^{*}=f\left( {\vec {\psi }_{a}^{*},\;F_{e}^{*}} \right)$$

where $\:f$ is the primary voltage’s relative function and $\:{\overrightarrow{V}}_{\text{a}}^{\text{*}}$ is the primary voltage reference. The dead-beat method is used to calculate the desired primary voltage in the next instant $\:{\overrightarrow{V}}_{\text{a},\:\text{k}+1}^{\text{*}}$ which uses the thrust and flux references rather than their predicted values, as expressed by

11 $$\vec {V}_{{a,~k+1}}^{*}=f\left( {\vec {\psi }_{a}^{*},F_{e}^{*}} \right)$$

The following stages serve as a brief summary of how the FS-PVC approach is implemented.

- Estimations of primary flux $\:{\psi\:}_{\alpha\:\beta\:a}$ as well as secondary flux $\:{\psi\:}_{\alpha\:\beta\:b}$, are elaborated by.

12 $${\psi _{\alpha \beta a}}^{{\left( k \right)}}=~{T_s}{u_{\alpha \beta a}}^{{\left( k \right)}} - {T_s}{R_a}{i_{\alpha \beta a}}^{{\left( k \right)}}+{\psi _{\alpha \beta a}}^{{\left( {k - 1} \right)}}~$$

13 $${\psi _{\alpha \beta b}}^{{\left( k \right)}}={T_l}\,{\psi _{\alpha \beta a}}^{{\left( k \right)}}+~\left( {{L_m} - \frac{{{L_a}{L_b}}}{{{L_m}}}} \right){i_{\alpha \beta a}}^{{\left( k \right)}}~$$

- Computation the primary voltage reference$\:\:{v}_{\alpha\:\beta\:p}^{*}$.

For the next control period, the predicted thrust is set to its reference value based on the deadbeat control approach. Likewise, the reference value determines the predicted primary flux value, as shown below

14 $$F_{{e\,(k + 1)}}^{*} = F_{e}^{*} = \frac{{3\pi }}{{2\tau \sigma }}\left( {\left| {\psi _{b} } \right|~ \cdot \left| {\psi _{a}^{*} } \right|\sin \theta ^{*} } \right)$$

15 $$\left| {\psi {{_{a}^{*}}_{\,\left( {k+1} \right)}}} \right|=\psi _{a}^{*}$$

The desired primary flux angle $\:{\theta\:}^{*}$ of the next period can be computed using

16 $${\theta ^*}={\sin ^{ - 1}}\left( {2\tau \sigma F_{e}^{*}/3\pi \psi _{a}^{*}{\psi _b}} \right)$$

17 $${\theta _{{\psi _b}}}=2{\tan ^{ - 1}}\left( {\frac{{{\psi _{qb}}^{{\left( k \right)}}}}{{{\psi _{db}}^{{\left( k \right)}}}}} \right) - {\tan ^{ - 1}}\left( {\frac{{{\psi _{qb}}^{{\left( {k - 1} \right)}}}}{{{\psi _{db}}^{{\left( {k - 1} \right)}}}}} \right)$$

18 $${\theta _{\psi _{a}^{*}}}={\theta _{{\psi _b}}}+{\theta ^*}$$

The secondary flux angle $\:{\theta\:}_{\psi\:b}$ is added to the calculate angle as obtained from the thrust equation in the rotating frame. So, the primary flux reference $\psi _{a}^{*}$can be calculated by

19 $$\overrightarrow {\psi _{a}^{*}} =\left| {\psi _{a}^{*}} \right|\exp \left( {j \cdot {\theta _{\psi _{a}^{*}}}} \right)$$

Both the predicted primary flux and the predicted flux angle can eventually obtain the desired *αβ*-axis voltage reference in the next control period, as determined by

20 $$V_{{\alpha \beta a\;\left( {k+1} \right)}}^{*}={T_s}^{{ - 1}}\psi _{{\alpha \beta a\;\left( {k+1} \right)}}^{*} - {T_s}^{{ - 1}}\psi _{{\alpha \beta a}}^{{(k - 1)}}+{R_a}{i_{\alpha \beta a}}$$

- Optimization on the cost function.

The error between the $\:{i}^{th}$ output VV of the inverter states and the primary reference voltage on the *αβ*-axis makes up the proposed cost function. The ideal choice for the next sample period will be the VV that minimizes the cost function’s (*J*) value (i.e., the least distance between the reference voltage and the candidates), where only the *αβ*-axis primary voltage tracking error between the predicted and their reference values are involved, as follows:

21 $${J_i}{\text{= }}\left( {{{\text{V}}_{{\text{\varvec{\upalpha}a}},{\text{i}}}} - {\text{V}}{{_{{{\text{\varvec{\upalpha}a}}\,}}^{{\text{*}}}}_{{\text{(k+1)}}}}} \right){\text{+}}\left( {{{\text{V}}_{{\text{\varvec{\upbeta}a}},{\text{i}}}} - {\text{V}}{{_{{{\text{\varvec{\upbeta}a}}}}^{{\text{*}}}}_{\,{\text{(k+1)}}}}} \right),\,i \to \,\left( {1,2,3,. \ldots ,8} \right)$$

**Fig. 3:**
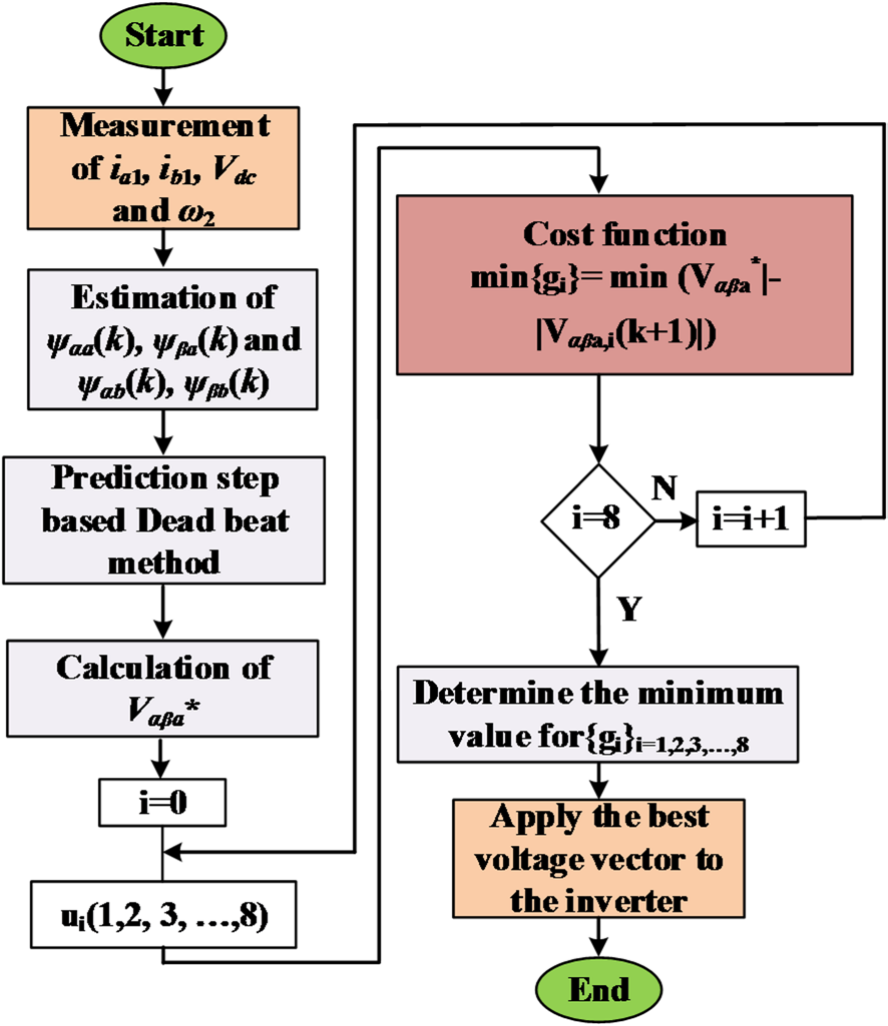
The flow chart of the FS-MPVC strategy.

Unlike the traditional prediction model^22^, which requires seven iterations during each control period, the proposed method relies on a single prediction using the deadbeat control concept. This results in a significantly shorter computation time. Figure 3 shows the flow chart of the FS-MPVC for LIM drive system.

## Proposed terminal sliding mode control based speed control with FS-MPVC for the LIM

The LIM’s speed control system is designed using terminal sliding mode control within the FS-PVC framework, according to the speed control loop for the LIM.

### Speed control utilizing sliding trajectory

The LIM speed error has been selected as the sliding mode state variable for the speed loop. The goal of the LIM speed controller is to make the LIM speed *v*_*b*_ follow the *v*_b_* reference speed. This objective can be achieved by the creation of the control law. To illustrate the discrepancy between the preset linear speed *v*_b_* and its actual value *v*_*b*_, the tracking error $\mu$ and its derivation $\dot {\mu }$ are provided as follows.

22 $$\left. {\begin{array}{*{20}{c}} {\mu =v_{b}^{*} - {v_b}} \\ {\dot {\mu }=\dot {v}_{b}^{*} - {{\dot {v}}_b}} \end{array}} \right\}$$

It is possible to define the terminal sliding mode trajectory as

23 $$s=\dot {\mu }+k*{\left| \mu \right|^\alpha }$$

where *k* is a constant gain and $0<\alpha <1$ .

The SMC design heavily relies on the reaching law, which is depicted as

24 $$\dot {s}= - {k_2}s - {k_1}sign(s){\text{ }}{k_1} \geqslant 0,{k_2}>0$$

where the constant parameters of the reaching law are denoted by the letters *k*_*1*_ and *k*_2_. The sliding mode state will be reached on the sliding surface with stability when the $s\dot {s}$ condition is met^9^.

### Speed regulation based on final control law

Developing a controller for the speed loop will allow the LIM’s actual signal to follow its reference value. As a result, the TSMC’s speed control loop is meticulously designed so that the actual speed stays relatively close to the LIM speed’s preset value. Therefore, using the TSMC approach and the mechanical Eq. (6), the speed control loop can be modified as indicated by

25 $${\dot {v}_b}={B_n}{F_e}+{D_n}{F_l}+{A_n}{v_b}$$

where *B*_*n*_*=-D*_*n*_*=(JG)*^−1^, and *A*_*n*_*=-H/G*, are different factors under the nominal working conditions. Moreover, *G* represents mass, and *H* is the viscous coefficient of LIM. Under external load disturbance, the LIM’s mechanical dynamics can be rewritten as

26 $${\dot {v}_b}=({B_n}+\Delta B){F_e}+({D_n}+\Delta D){F_l}+({A_n}+\Delta A){v_b}$$

27 $${\dot {v}_b}={B_n}{F_e}+\Delta P$$

In the meanwhile, the $\Delta P(t)$ can be written as

28 $$\Delta P= - ({D_n}+\Delta D){F_l} - ({A_n}+\Delta A){v_b} - \Delta B({F_e}^{ * } - {F_e})$$

By replacing (27) into the derivative of speed error ($\dot {\mu }={\dot {v}_b}^{*} - {\dot {v}_b}$), it will get

29 $$\dot {\mu }={\dot {v}_b}^{*} - {\dot {v}_b}={\dot {v}_b}^{*} - {B_n}{F^ * }_{e} - \Delta P(t)$$

The sliding mode surface and reaching law are defined in (23) and (24), separately. By combining (23), (24), and (29), the thrust reference based on the TSMC can be expressed as

30 $$\begin{gathered} {F_e}^{{^{*}}}={B_n}^{{ - 1}}\,\left( {k*{{\dot {v}}_r}^{*}+{{\ddot {v}}_r}^{ * } - {A_n}k*{{\left| \mu \right|}^\alpha }} \right. \hfill \\ \,\,\,\,\,\,\,\,\,\,\,\,\,\,\,\,\,\,\,\,\,\left. {+\int\limits_{0}^{t} { - {k_1}s - {k_2}sign(s){\text{ }}d\gamma } } \right) \hfill \\ \end{gathered}$$

The sliding mode state will arrive at the recommended sliding trajectory in a finite amount of time with zero error since the control law’s coefficients, *k1*, *k2*, and *k*, were carefully chosen. The use of the discontinuous sign function, *sgn(s)*, often leads to significant chattering and undesirable system behavior. To mitigate this issue, the continuous saturation function, *sat(s)*, is employed as a replacement. This function effectively reduces chattering and can be expressed as follows

31 $$\:sat\left(s\right)=s/(s+\gamma\:)$$

**Fig. 4:**
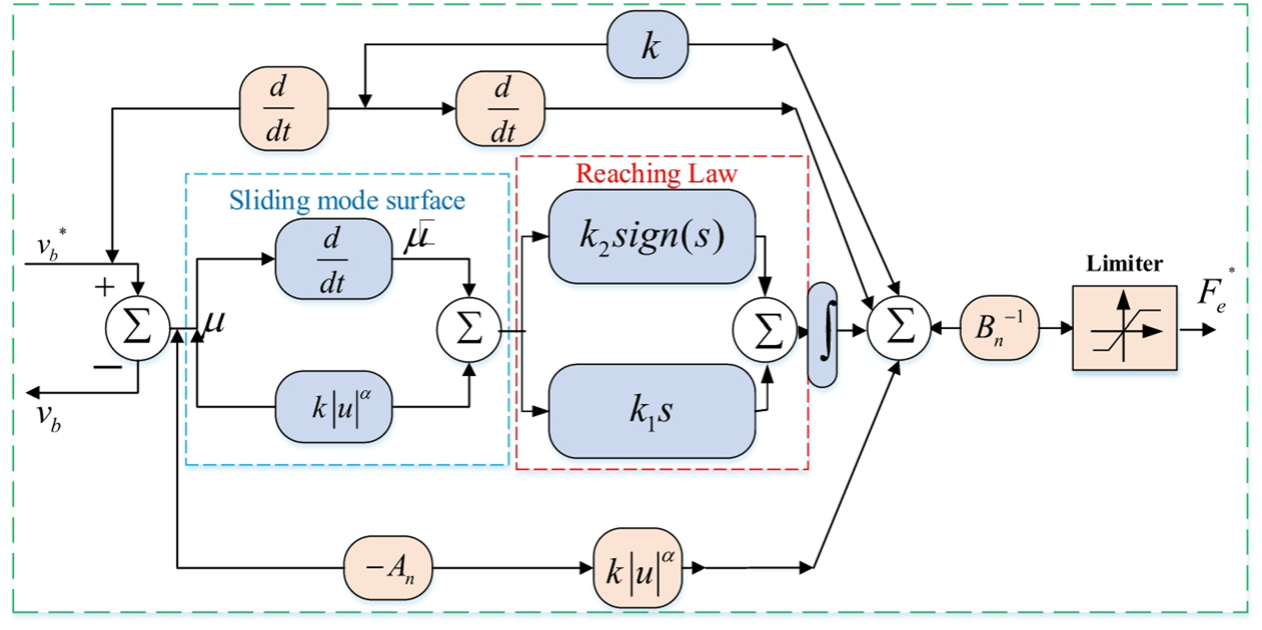
The reference thrust of LIM speed control based TSMC method.

**Fig. 5:**
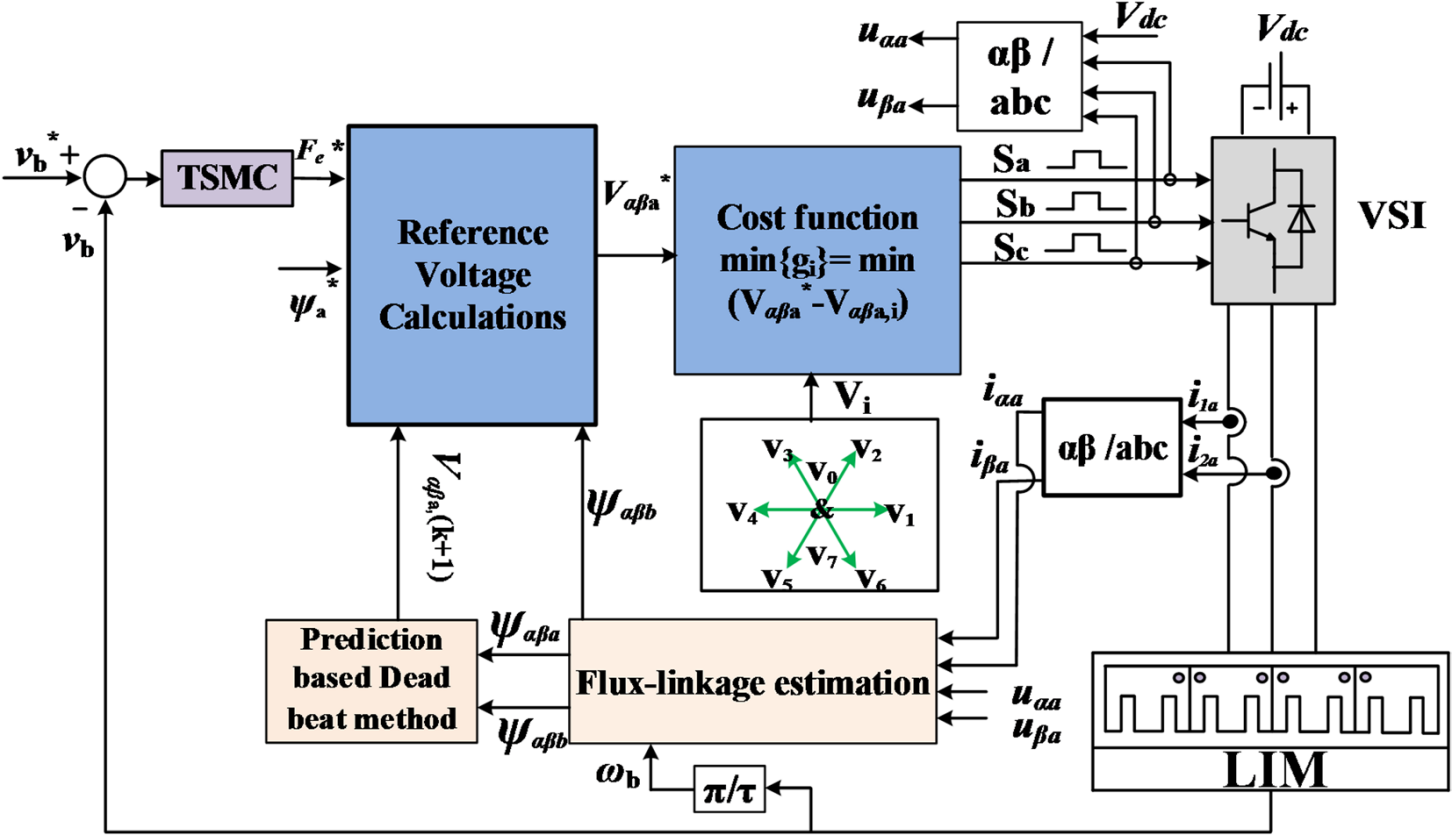
The TSMC based FS-MPVC for the LIM drive system.

Where $\:\gamma\:$ is a small positive constant. The LIM system based on the TSMC method for is presented in Fig. 4. Moreover, the final block diagram of the TSMC with FS-MPVC is depicted in Fig. 5.

## Simulation and analysis

To verify that the proposed TSMC based on the FS-PVC method is superior and effective. The linear speed, thrust, primary flux and primary current of the LIM under TSMC-MPVC, CSMC-MPVC and PI-MPVC control methods are compared through simulation analyses under three operating scenarios. The machine and the simulation parameters are defined in Tables 2 and 3, respectively.

### Case 1: Start-up response

**Fig. 6:**
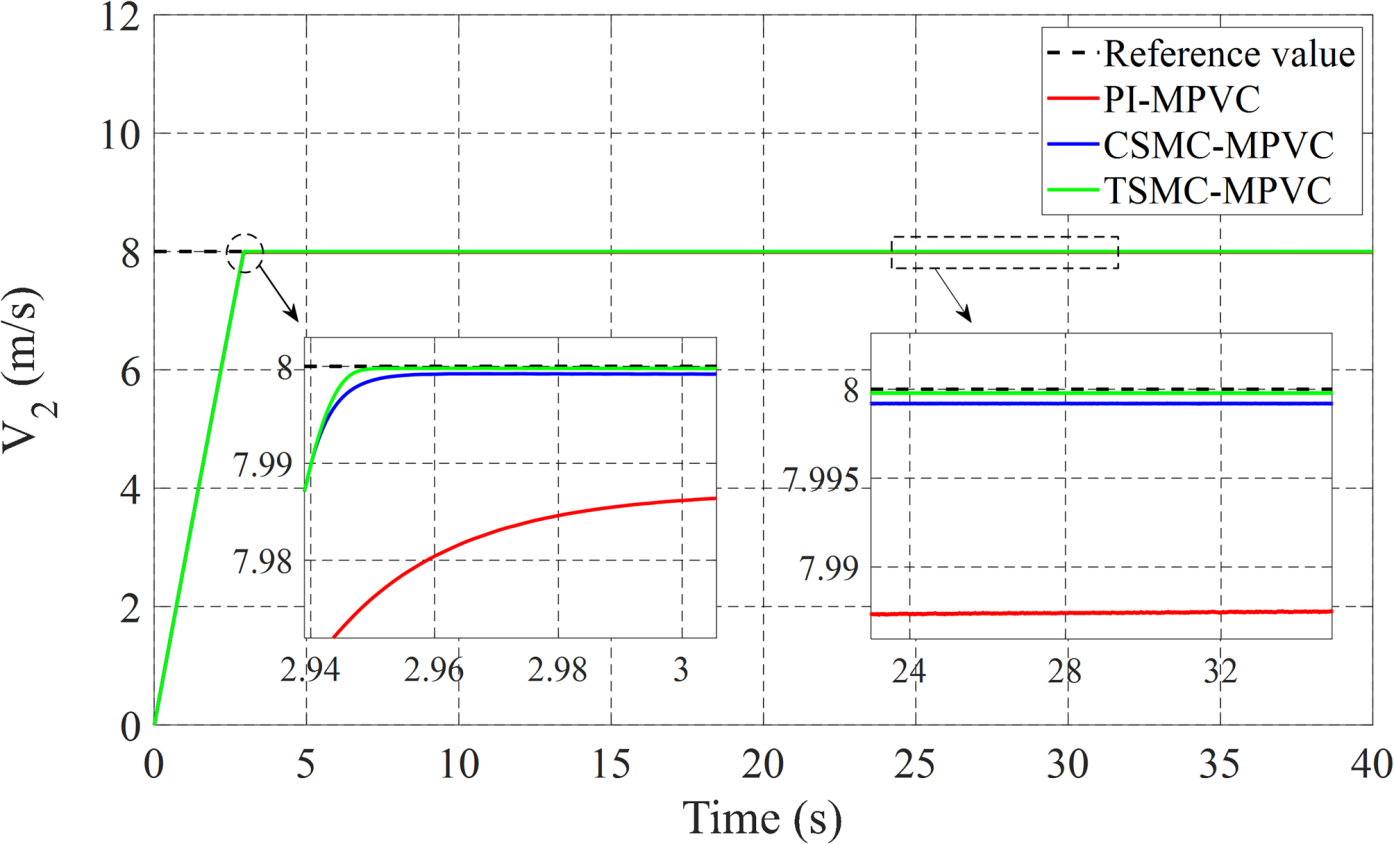
Speed response of all control strategies under starting process.

**Fig. 7:**
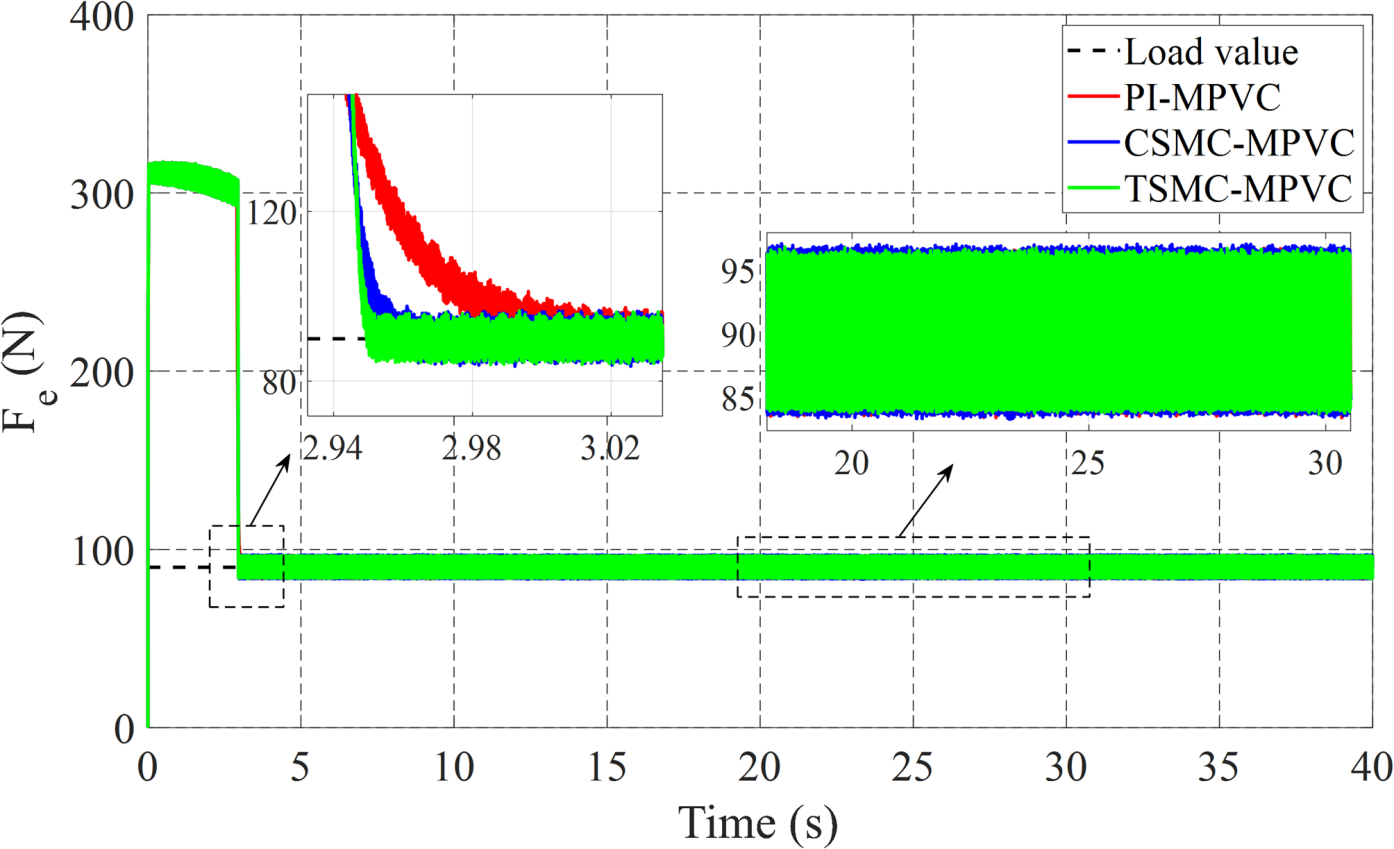
Thrust response of all control strategies under starting process.

**Fig. 8:**
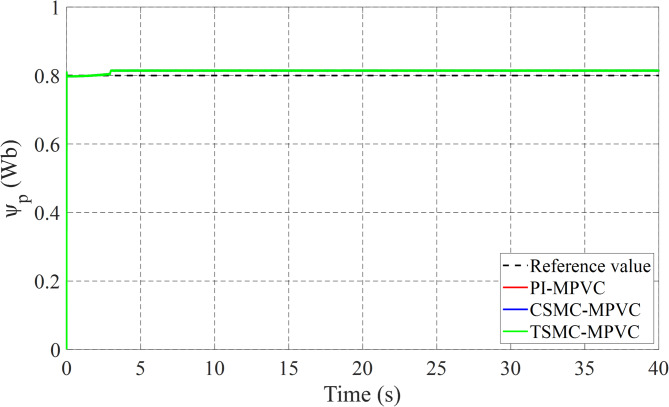
Primary flux of all control strategies response under starting process.

**Fig. 9:**
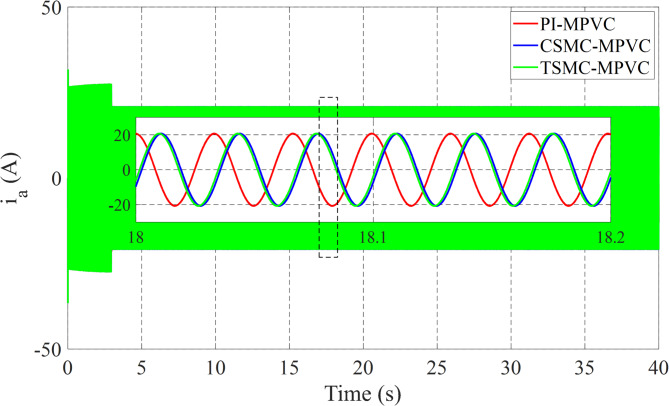
Input Phase-A response of all control strategies under starting process.

The performance of three test methods for this case has been evaluated under specific working conditions. In the assessment, constant load and speed responses were examined on the LIM drive system. The speed response was varied from 0 to 8 m/s with a 90 N load maintained for the entire simulation duration across TSMC-MPVC, CSMC-MPVC, and PI-MPVC methods individually. Upon analysis, it is evident from Fig. 6 that the speed response achieved with TSMC-MPVC exhibits superior tracking capability with faster convergence compared to CSMC-MPVC and PI-MPVC methods. This suggests the efficacy of the TSMC-MPVC approach over its counterparts in achieving desired speed responses efficiently. Furthermore, Fig. 7 illustrates the thrust results of the LIM drive system. It is noteworthy that the thrust attained through TSMC-MPVC method exhibits quicker initiation towards reaching steady-state operating conditions compared to CSMC-MPVC and PI-MPVC methods, respectively. This enhancement underscores the superior performance of the TSMC-MPVC method in terms of both speed response tracking and thrust attainment, indicating its effectiveness in practical applications over the other evaluated methods. Moreover, Fig. 7 reveals that the proposed TSMC-MPVC method exhibits the smallest thrust ripple value as well as reaches steady-state performance more rapidly compared to the CSMC-MPVC and PI-MPVC control methods. This reduction in thrust ripple signifies enhanced stability and smoother operation, highlighting the superiority of the TSMC-MPVC approach. Additionally, Fig. 8 presents the primary flux results for all control techniques, indicating nearly identical values during the specified working conditions. This suggests that all methods are comparable in terms of primary flux performance under these conditions. Finally, Fig. 9 depicts the phase current during the starting performance phase for TSMC-MPVC, CSMC-MPVC, and PI-MPVC methods. It is evident from Fig. 9 that the starting current time for TSMC-MPVC is significantly lower than that of CSMC-MPVC and PI-MPVC methods, respectively. This reduction in starting current time further emphasizes the efficiency and responsiveness of the TSMC-MPVC method, making it a favorable choice for applications requiring quick and precise control responses.

**Table 2:** Detailed parameters of the AIM.

Quantity	Symbol	Value	Unit
Rated thrust	*F*_*N*_	270	N
Rated speed	*v*_*N*_	11	m/s
Rated current	*I*_*N*_	22	A
Rated voltage	*v*	180	V
Primary length	*l*_*s*_	1.3087	m
Primary leakage inductance	*L*_*la*_	0.009	H
Primary resistance	*R*_*a*_	1.06	Ω
Secondary resistance	*R*_*b*_	2.4	Ω
Pole pitch	*τ*	0.1485	m

**Table 3:** The control methods parameters.

Name	Value
PI gains for PI-MPVC	300 and 0.3
Switching gains for the TSMC-MPVC	$\mu =300,{\text{ }}{k_1}=150,{\text{ }}{k_2}=10$

### **Case 2**: speed change response

**Fig. 10:**
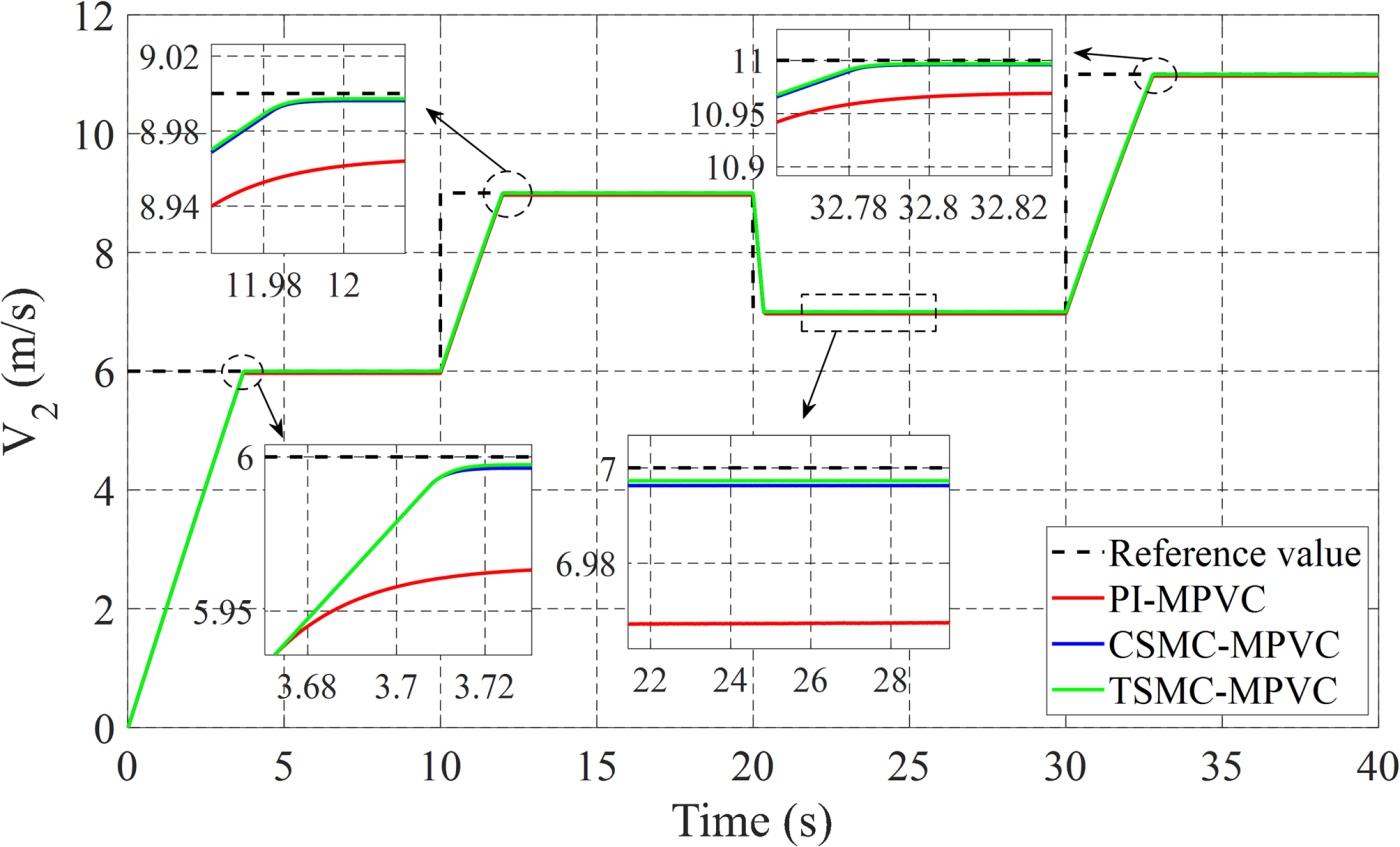
Speed response under variable speed.

**Fig. 11:**
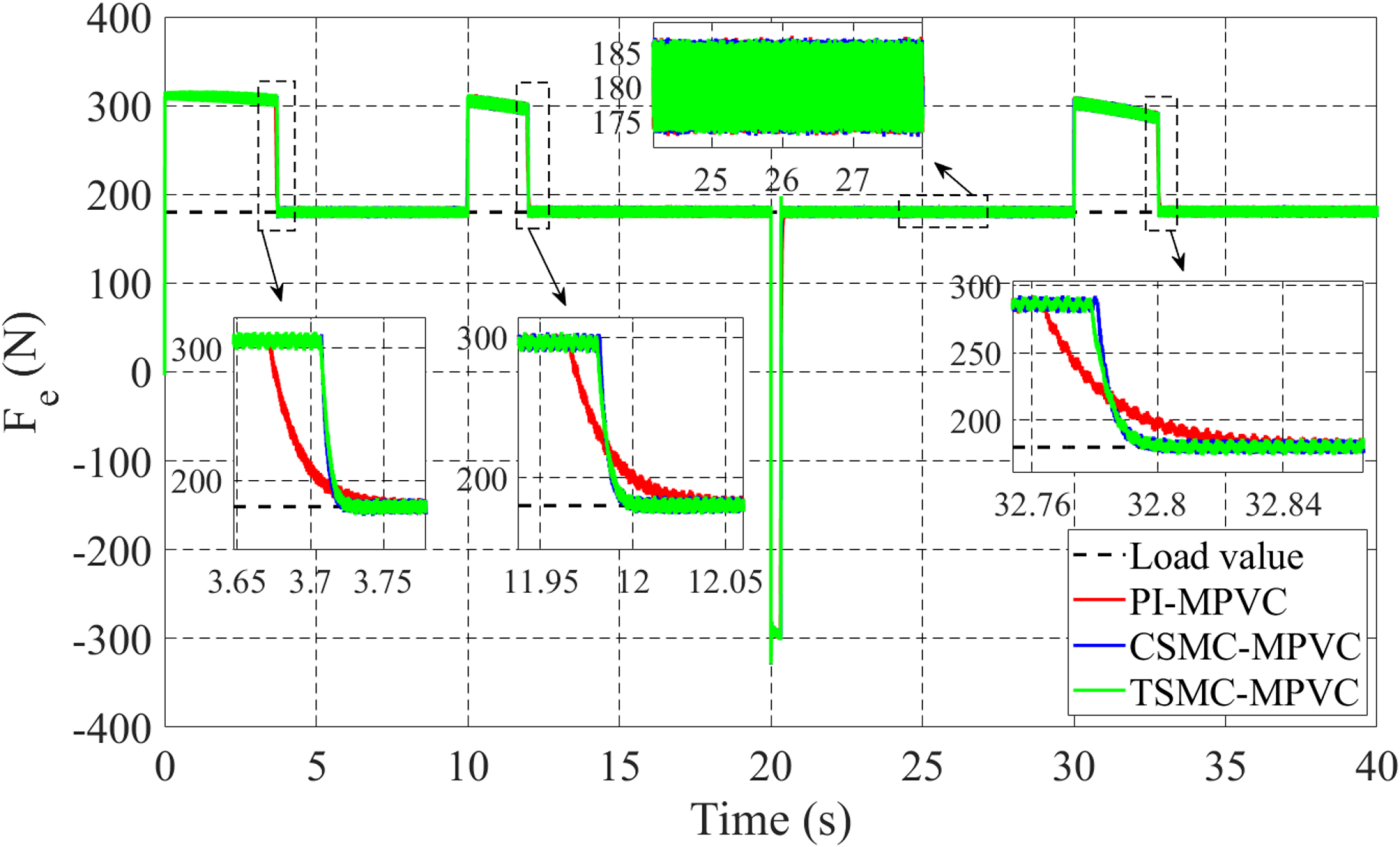
Thurst response under variable speed.

The linear speed change response on the LIM drive system has been fully checked in this subsection under TSMC-MPVC, CSMC-MPVC, and PI-MPVC separately. The working conditions of three test methods are set as follows: A constant load of 180 N on LIM is applied during the total simulation time. The reference speed 6 m/s is set for 0–10 s. During 10–20 s, set the reference speed 9 m/s. During 20–30 s, set the reference speed 7 m/s. During 30–40 s, set the reference speed 11 m/s has been depicted in Fig. 10. It can be observed adopting this working scenario that the behavior of the speed with TSMC-MPVC has a short period for reaching its reference as well as a smaller tracking error than that of CSMC-MPVC and PI-MPVC. Moreover, Fig. 11 shows the thrust response under this working scenario for TSMC-MPVC, CSMC-MPVC, and PI-MPVC, respectively. Compared with the PI-MPVC and CSMC-MPVC, TSMC-MPVC has a faster response to reach steady-state behaviors.

**Fig. 12:**
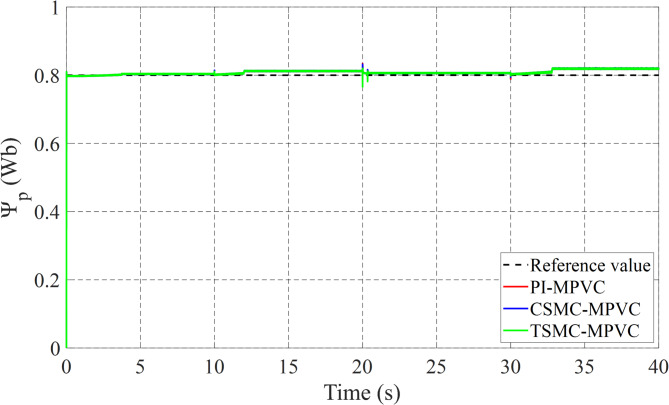
Primary flux-linkage response under variable speed.

**Fig. 13:**
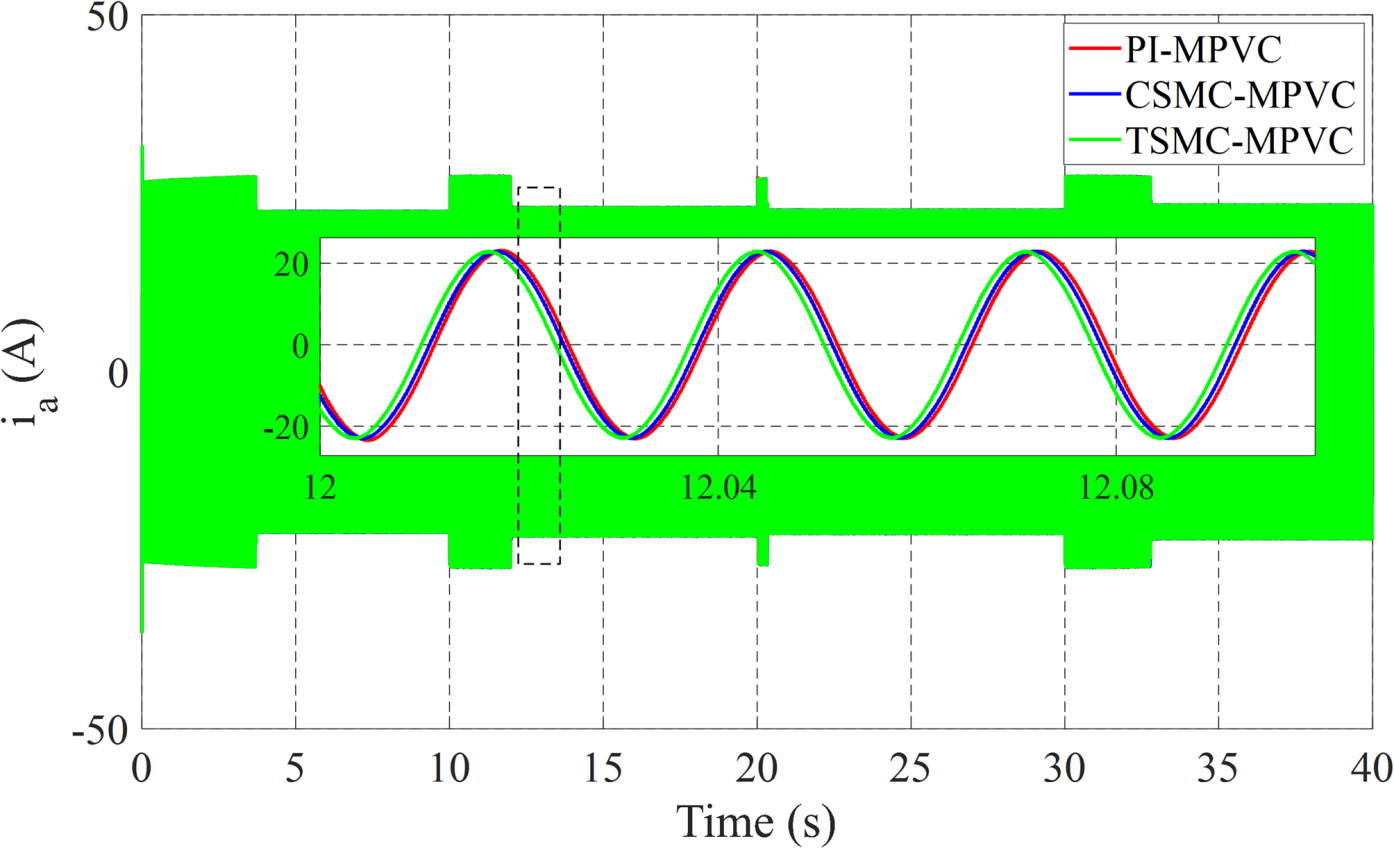
Input Phase-A current response under variable speed.

Figure 12 shows the primary flux result for all control techniques under this working condition. It can be noted that the actual values are nearly the same during this working case. Figure 13 shows the results of the input Phase-A current with TSMC-MPVC, CSMC-MPVC, and PI-MPVC. It can be seen from this result that the current reaches a set rated value for a short period under the TSMC-MPCC than that of other control methods.

### Case 3: load change response

**Fig. 14:**
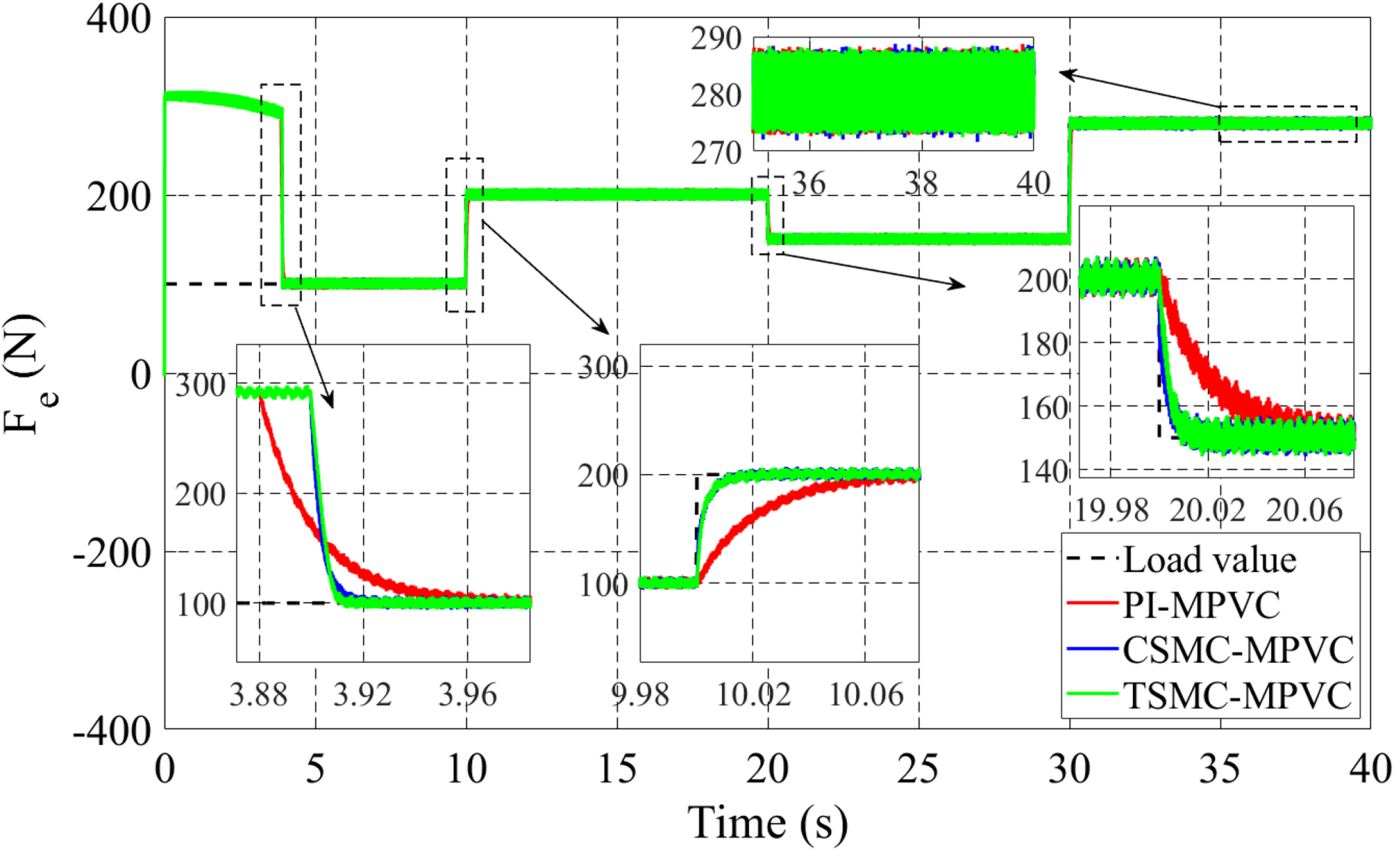
Thrust response under variable load.

**Fig. 15:**
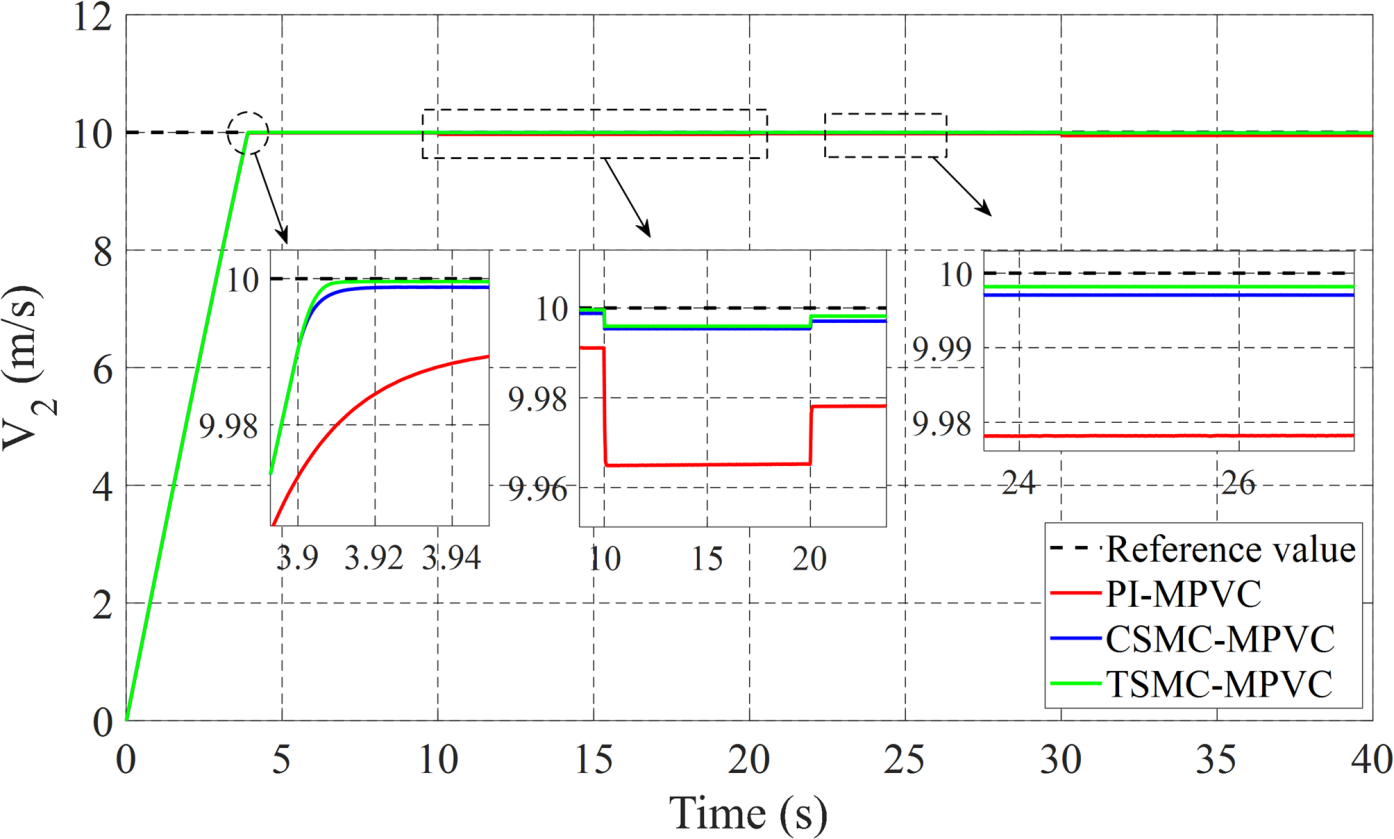
Speed response under variable load.

**Fig. 16:**
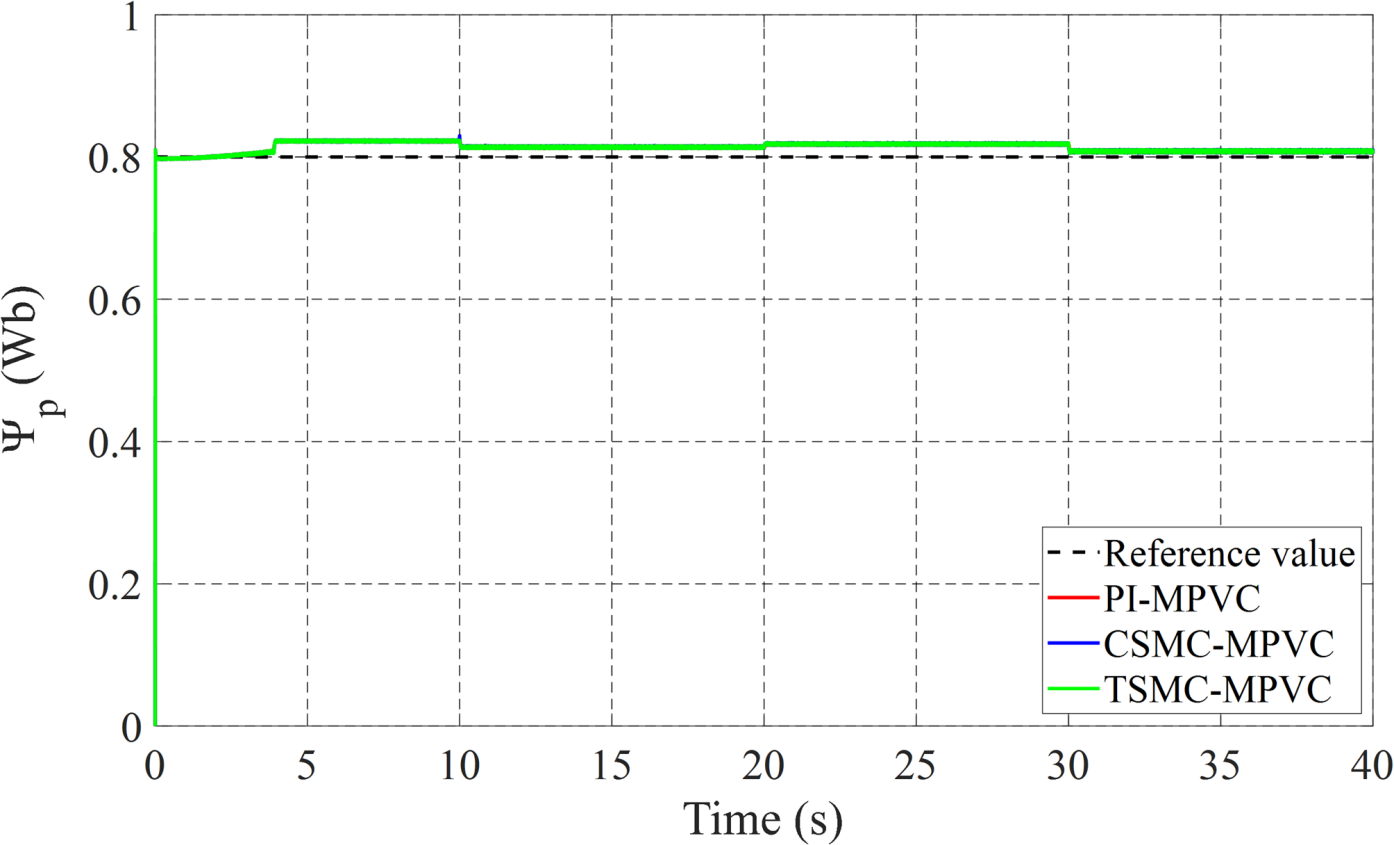
Primary flux under variable load.

**Fig. 17:**
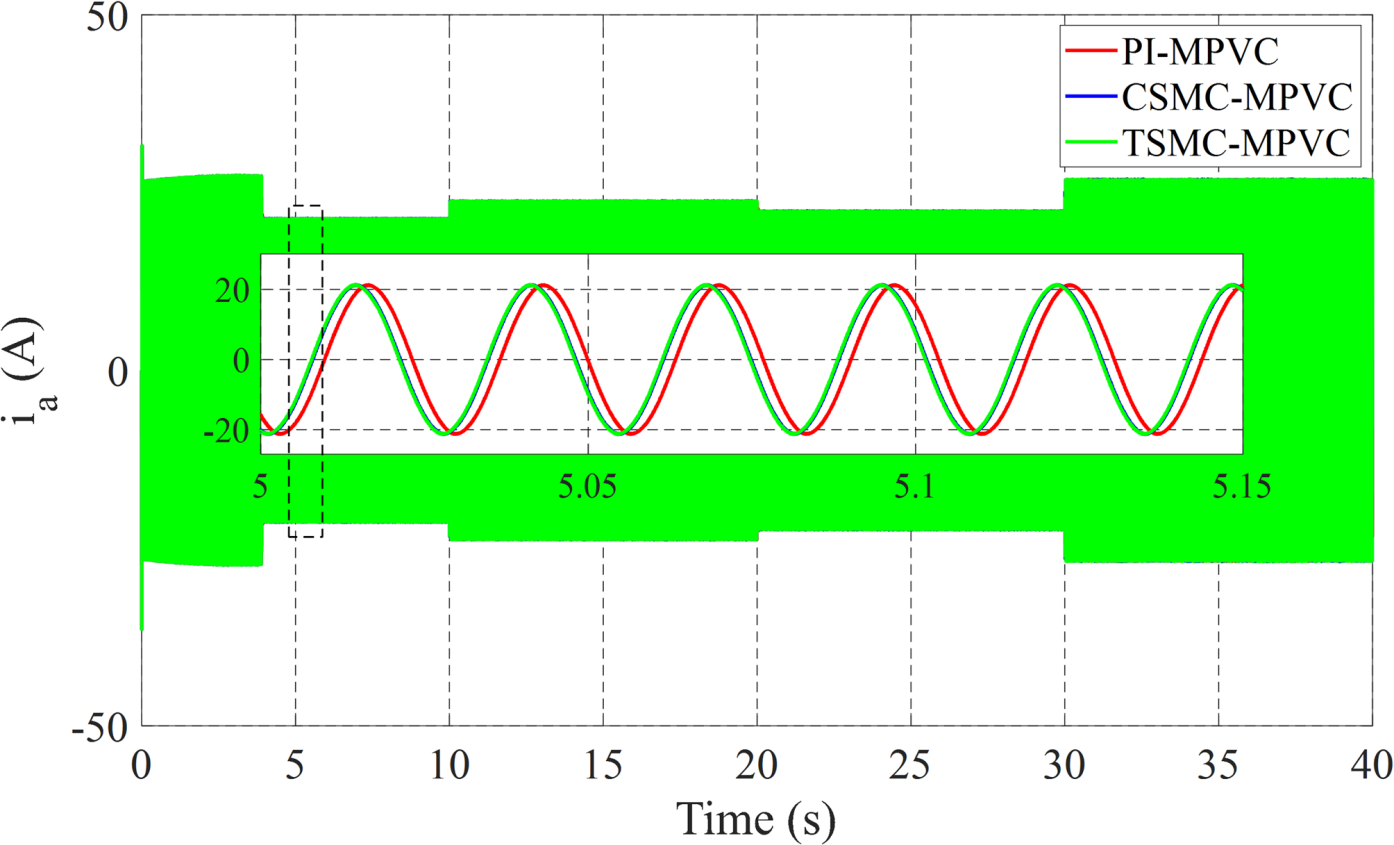
Input Phase-A current under variable load.

The load change dynamic response test under constant speed is fully studied in this subsection under TSMC-MPVC, CSMC-MPVC, and PI-MPVC separately for the LIM drive system. Firstly, the LIM drive has been started when machines reach the steady state condition, hence a 100 N load has been added and the load has been increased by 100% from 100 N to 200 N during 10–20 s with a reference speed set as 10 m/s. Again, the load has been reduced to the value, 150 N during 20-30s. Afterwards, the load has been increased from 150 N to 280 N during 30–40 s. Figure 14 shows the thrust performance under the three control methods thorough this simulation scenario. From this figure, it can be observed that the response has faster convergence with TSMC-MPVC than those with CSMC-MPVC and PI-MPVC, individually. This further confirms the superiority of the proposed TSMC-MPVC method. The speed response for different control methods is compared and presented in Fig. 15. It is depicted that the speed response with TSMC-MPVC has a short period for reaching its reference value as well as a smaller tracking error than that of CSMC-MPVC and PI-MPVC, which proves the effectively the proposed TSMC-MPVC method. The proposed method responds more quickly response, because the TSMC can enter sliding mode states very fast when it is near or far from the designed surface with robust anti-disturbances. Similar to the starting case, the primary flux linkage performance when the load changed for TSMC-MPVC, CSMC-MPVC, and PI-MPVC is shown in Fig. 16. It can be concluded from this image that the actual values are nearly the same during this working case for different control methods. Moreover, it is demonstrated that the Phase-A current is effectively changed in different working loads for three control methods with a short period for reaching a set rated value under the proposed TSMC-MPVC method as presented in Fig. 17.

## Conclusions

To enhance the dynamic response of the LIM drive system, this study introduces a FS-MPVC method based on TSMC. The proposed MPVC simplifies the tuning complexity of the balancing coefficient by constructing its cost function based on the primary voltage. Additionally, TSMC is fully integrated with a speed loop using the MPVC approach. The three control methods, proposed TSMC-MPVC, CSMC-MPVC and PI-MPVC were compared under various operating conditions, including startup, load changes, and speed alteration. Overall, the simulation results demonstrate that the TSMC-MPVC method outperforms the other methods in terms of effectiveness and features. Notably, TSMC-MPVC reduces thrust and flux ripples, thereby effectively minimizing the LIM’s noise and vibration. The proposed scheme should be validated through experimental tests in subsequent work.

## Data Availability

All data generated or analysed during this study are included in this published article.
